# Acute NO_2_ Stress Shortens the Median Survival Period of *Bougainvillea glabra* ‘Elizabeth Angus’ by Disrupting Tissue Structure and Photosynthetic Response Centers

**DOI:** 10.3390/plants12234028

**Published:** 2023-11-30

**Authors:** Yuxiang Liang, Xinchen Qian, Shuang Song, Qianqian Sheng, Zunling Zhu

**Affiliations:** 1 College of Landscape Architecture, Nanjing Forestry University, Nanjing 210037, China; liangyuxiang@njfu.edu.cn (Y.L.);; 2 The Center of Southern Modern Forestry Cooperative Innovation, Nanjing Forestry University, Nanjing 210037, China; 3 Research Center for Digital Innovation Design, Nanjing Forestry University, Nanjing 210037, China; 4 Jin Pu Research Institute, Nanjing Forestry University, Nanjing 210037, China; 5 College of Art and Design, Nanjing Forestry University, Nanjing 210037, China

**Keywords:** NO_2_ stress, *Bougainvillea*, apparent traits, physiological characteristics, K-M survival curve

## Abstract

The air pollutant NO_2_ is one of the major constraints on plant growth, and the ecological value of the ornamental plant *Bougainvillea glabra* can be weakened by NO_2_. In this study, an indoor 4 μL·L^−1^ NO_2_ simulated fumigation test was conducted with three treatments, CK (normal growth with clean air), T1 (4 μL·L^−1^ NO_2_ + 8 h/d), and T2 (4 μL·L^−1^ NO_2_ + 24 h/d), which were set up with considerations for time and concentration. The results demonstrated that most of the morphological parameters of *B. glabra* ‘Elizabeth Angus’, except for the floral organs, were decreased in the root, stem, leaf, and bract. Continuous fumigation significantly attenuated the growth rate and reduced the water and pigment contents of organs. Excessive NO_2_ reduced the number and transfer rate of photoelectrons by destroying the photosynthetic reaction center, which in turn weakened photosynthesis, but the plants with intermittent fumigation recovered after fumigation. The Kaplan-Meier (K-M) survival curve displayed median survival periods of 41 and 55.5 h for T1 and T2, respectively, and the morphological structure and most of the indicators of photosynthetic reaction centers changed significantly during stress. Acute injury to *B. glabra* ‘Elizabeth Angus’ was caused by 4 μL·L^−1^ NO_2_, and *B. glabra* ‘Elizabeth Angus’ had limited ability to regulate high concentrations of NO_2_ acute stress.

## 1. Introduction

Air plays a crucial role in maintaining the normal physiological activities of plants and is regarded as one of the fundamental prerequisites for their growth and development. However, the expansion of urban and industrial areas in recent years has resulted in excessive emissions of air pollutants, primarily including sulfur dioxide, nitrogen oxides, and particulate matter, which have had significant adverse impacts on plant life. Among these pollutants, nitrogen dioxide (NO_2_) is emitted by motor vehicles and industrial production, and it is characterized by its stable chemical structure, high proportion, and harmful effects on plants among all nitrogen oxides [1]. Its derivatives can easily contribute to haze and acid rain formation in certain environmental conditions [2]. Exposure to NO_2_ can lead to various morphological and metabolic disorders in plants, including woody plants, thus impacting their normal growth and development [3,4,5]. Therefore, it is crucial to comprehend the toxic mechanism of NO_2_ on plants and how plants can resist NO_2_ damage in the field of plant resistance.

The response mechanism of plants to NO_2_ is relatively complex. Research has found that low concentrations (40–60 nL·L^−1^) positively affect plant growth, while medium to high concentrations (1–4 μL·L^−1^) have a negative effect. In general, changes in plant surface traits are often caused by specific gene expression levels and metabolic activity changes within the plant [6,7]. Although there is significant heterogeneity among different plants [3], there is a strong correlation between physiological changes in plant morphology and overall NO_2_ stress. Apparent traits and anatomical structure are important indicators to measure the degree of damage [8,9], such as the degrees of deformation and decomposition observed in the cellular morphology and structure of plant organs, particularly leaves, under NO_2_ stress [10]. In addition, other organs also exhibit varying degrees of damage in the NO_2_ environment, such as root atrophy [11], lodging caused by decreased stem strength [12], etc. Additionally, plant damage is associated with physiological changes, specifically in photosynthesis, which is evident in decreased chlorophyll content and increased water loss. The above phenomenon has been verified in various plants, such as *Arabidopsis thaliana* [13], *Betula platyphylla* [14], and *Carpinus putoensis* [15].

Plants’ morphological and physiological changes can be utilized to analyze the degree of damage under NO_2_ stress and the comprehensive changes at different time scales. Scholars have conducted a series of meaningful explorations in studying the damage level of NO_2_ on different plants. For instance, researchers have examined semi-lethal thresholds for various plants and plant combinations, as well as differences in resistance between indoor and outdoor green spaces, through techniques such as spectroscopy, mass spectrometry, and nuclear magnetic resonance (NMR) [16,17]. Furthermore, research has also been conducted on morphological responses [18] and metabolite regulation [19]. However, there is still room for improvement in the systematic analysis and evaluation of NO_2_ resistance based on apparent traits and physiological responses when studying the resistance response to a specific plant or plant group.

*Bougainvillea* spp. is a perennial evergreen shrub belonging to the *Bougainvillea* genus in the *Nyctaginaceae* family. It is extensively cultivated in urban landscaping projects in tropical and subtropical regions due to its exceptional ornamental value and diverse potential applications. *Bougainvillea* has several garden advantages, including rapid growth, high organ differentiation [20], and easy reproduction [21,22]. In addition, in terms of ecological regulation, *Bougainvillea* has a certain tolerance and regulation ability to water bodies [23,24], air [7,25,26], and soil [27]. It is an ideal plant for studying NO_2_ pollution under laboratory-sealed fumigation conditions. At the same time, *Bougainvillea* also faces the negative impact of increasing NO_2_ in industrial and mining areas where it grows. Our research group has conducted extensive studies on the response of NO_2_ in *Bougainvillea* from 0 to 8 μL·L^−1^ different concentrations. The results indicated that under medium concentrations of 4 μL·L^−1^, *Bougainvillea* showed noticeable signs of injury without resulting in mortality [7]. However, the differences and specific injury mechanisms under different time treatments remain unclear. Therefore, further exploration is needed to understand the specific response mechanism of *Bougainvillea* to NO_2_, along with conducting systematic research and comprehensive evaluation.

Building upon previous research conducted by the research group, this article systematically examines the response mechanisms of *Bougainvillea* to NO_2_. The study utilized a well-sealed experimental device to control and monitor NO_2_ concentration through a laboratory simulation fumigation test at a concentration of 4 μL·L^−1^ [7]. The investigation comprehensively explores the effects of NO_2_ on *Bougainvillea* by analyzing apparent traits, morphology and anatomical structure, water physiology, photosynthetic pigment content, chlorophyll fluorescence, and photosynthetic physiology. Furthermore, principal component analysis (PCA), membership function, and K-M survival curve were used to comprehensively evaluate the changes in various indicators under different treatments. The results of this study will further enrich the theory of plant reduction of atmospheric pollutants, expand the field of plant environmental ecotoxicology, and provide a theoretical basis for the targeted improvement of garden plant resistance and the application of NO_2_ management.

## 2. Results

### 2.1. Appearance, Anatomical Structure, and Morphological Indicators

#### 2.1.1. Changes in NO_2_ Concentration and Overall Plant Morphology

**Figure 1:**
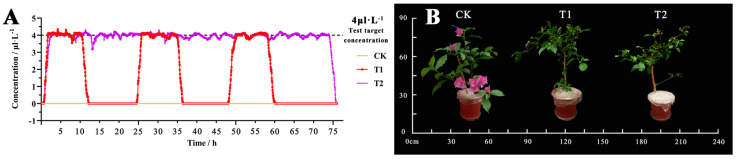
Gas concentration and plant changes under different concentration treatments. Note: CK, clean air; T1, 4 μL·L^−1^ (8 h/d); T2, 4 μL·L^−1^ (24 h/d). (**A**) Changes in NO_2_ concentration within 72 h of the fumigation box during the experimental period (data source: cloud platform monitoring data). (**B**) Changes in overall morphology of *B. glabra* ‘Elizabeth Angus’.

The data monitored under the cloud platform indicate that T1 (4 μL·L^−1^ NO_2_ + 8 h/d) and T2 (4 μL·L^−1^ NO_2_ + 24 h/d) maintained a stable concentration throughout the experiment (Figure 1A). The overall impact of NO_2_ treatment with different fumigation times on the planting of *B. glabra* ‘Elizabeth Angus’ is significant. Before treatment, the leaves of each group of plants thrived, with bracts growing at the top of the branches. After fumigation treatment, compared to T1 and T2 fumigation treatments, the leaves and flowers of the CK control group exhibited a strong growth status, showing no significant signs of leaf damage or flower loss. Additionally, the branches remained robust. T1 and T2 showed varying degrees of defoliation and flower shedding, and the leaves also showed varying degrees of damage (Figure 1B).

#### 2.1.2. Changes in Root Appearance, Anatomical Structure, and Morphological Indicators

**Figure 2:**
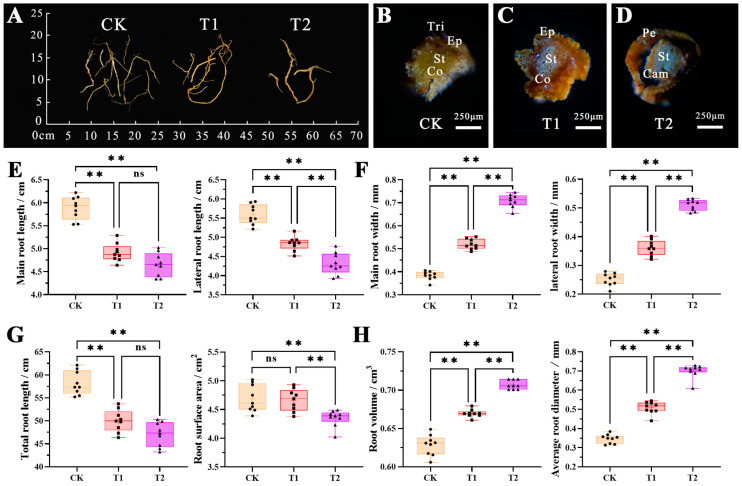
Changes in root appearance, anatomical structure, and morphological indicators of *B. glabra* ‘Elizabeth Angus’. (**A**) Changes in root appearance under different treatments. (**B**) Changes in root transverse structure of CK. (**C**) Changes in root transverse structure of T1. (**D**) Changes in root transverse structure of T2. Note: Tri. trichomes; Ep. epidermis; St. stele; Co. cortex; Pe. periderm; Cam. Cambium. Scale bar = 250 μm. (**E**) Main and lateral root length. (**F**) Main and later root width. (**G**) Root total length and surface area. (**H**) Root volume and average root diameter. Note: ** indicates highly significant differences in the same indicator between different treatments (*p* < 0.01); ns indicates that no significant difference in the same indicator between different treatments (*p* > 0.05). This applies to the rest of the text hereafter.

Roots are important plant organs that absorb nutrients such as water, gas, and inorganic salts. The duration of fumigation at a concentration of 4 μL·L^−1^ had different effects on the root system of *B. glabra* ‘Elizabeth Angus’. In the CK control group, the root system remained intact after fumigation (Figure 2A), and the main and lateral roots were milky white with a plump structure. Microscopic analysis revealed epidermal hairs around the root system, and the epidermis was smooth. The transverse structure presented a more obvious three-layer “epidermis-cortex-middle column” structure, showing a tendency for further growth (Figure 2B). However, in T1 and T2, the epidermal hairs decreased, the main and lateral roots turned yellow, and some structures were partially damaged. Both the stele and cortex showed a state of developmental arrest, particularly in T2, where gaps appeared between the forming layer and the periderm, resulting in severe overall structural damage, with continuous cork formation in the stele (Figure 2C,D).

The duration of fumigation at a concentration of 4 μL·L^−1^ caused significant alterations in the morphological parameters of the root system of *B. glabra* ‘Elizabeth Angus’. As shown in the figure, except for the root surface area of CK and T1, there was a significant difference among the treatment groups. Moreover, the length of the main and lateral roots showed a consistent and notable decreasing trend with increasing concentration and treatment time, and the differences between the groups were statistically significant (Figure 2E). The differences in root volume, average root diameter, and main lateral root thickness indexes (Figure 2F) among treatment groups were highly significant and showed an increasing trend with treatment time. In contrast, the total root length and root surface area of *B. glabra* ‘Elizabeth Angus’ exhibited a declining trend with increasing treatment concentration and duration (Figure 2G). The variations in root volume and average root diameter exhibited an inverse relationship with the variations in root length (Figure 2H). The above results indicated that exposure to NO_2_ increased root thickness and volume while causing a decrease in root length.

#### 2.1.3. Changes in Stem Appearance, Anatomical Structure, and Morphological Indicators

**Figure 3:**
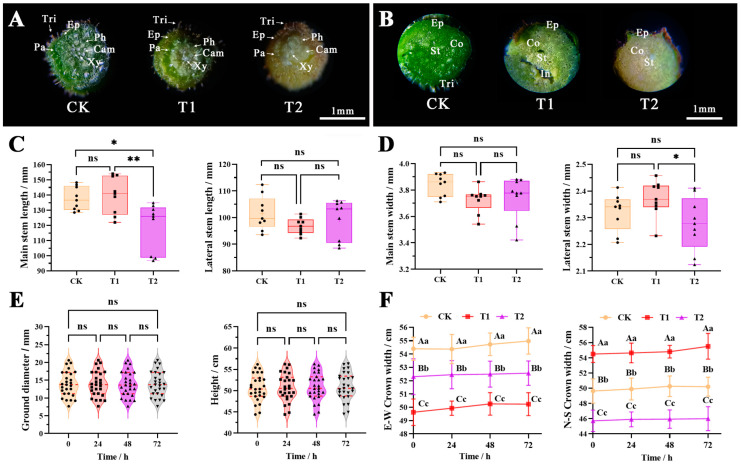
Changes in stem appearance, anatomical structure, and morphological indicators of *B. glabra* ‘Elizabeth Angus’. (**A**) Petiole transverse structure under an optical microscope. (**B**) Stem transverse structure under an optical microscope. Note: Tri. trichomes; Ep. epidermis; Co. cortex; St. stele; Pa. parenchyma; Ph. Phloem; Cam. Cambium; Xy. xylem. Scale bar = 1 mm. (**C**) Main and lateral stem length. (**D**) Main and lateral stem width. (**E**) Ground diameter and height (sample data from CK T1 and T2 simultaneously). (**F**) E-W and N-S crown width Note: The values are means ± SD (*n* = 3); ns indicates that no significant difference in the same indicator between different treatments (*p* > 0.05); The differences were compared by Duncan’s test, capital letters and ** indicate highly significant differences in the same indicator between different treatments (*p* < 0.01); small letters and * indicate significant differences in the same indicator between different treatments (*p* < 0.01). This applies to the rest of the text hereafter.

Stems are vital plant structures that connect roots and leaves, serving as crucial organs for transporting nutrients and providing morphological support. Figure 3 illustrates that exposure to stress caused a change in leaf petiole color, transitioning from green to yellow (Figure 3A). Moreover, there were varying degrees of reduction in chlorophyll and water content, accompanied by a gradual decrease in petiole diameter. The differentiation of tissue structure became less apparent, and certain structures such as epidermal hairs, xylem, and forming layer showed a decrease in quantity or insufficient differentiation. Significant changes were illustrated in the stems of *B. glabra* ‘Elizabeth Angus’ under different fumigation time treatments at 4 μL·L^−1^ concentration (Figure 3B). Compared with the CK control group, the number of skin hairs on the outer surface of the stem decreased in T1 and T2. Moreover, the color of the transverse structures was partially weakened, and there were noticeable gaps between the structures. Overall, the stress group’s stem transport function was weaker than the CK control group. These findings indicated that the functional and structural stability of *B. glabra* ‘Elizabeth Angus’ petioles was negatively correlated with the treatment times.

The figure shows that the length of fumigation time at a concentration of 4 μL·L^−1^ had a minor effect on the morphological parameters of stems. Except for the length of the main stem (Figure 3C), the lateral stem length, main lateral stem thickness, ground diameter, plant height, and north–south crown width did not differ significantly among the different concentration treatments (Figure 3D–F). Compared with the CK, NO_2_ stress resulted in a reduced growth rate in the main lateral stems of *B. glabra* ‘Elizabeth Angus’. However, it did not significantly impact the overall growth status of the branches.

#### 2.1.4. Changes in Leaf Appearance, Anatomical Structure, and Morphological Indicators

**Figure 4:**
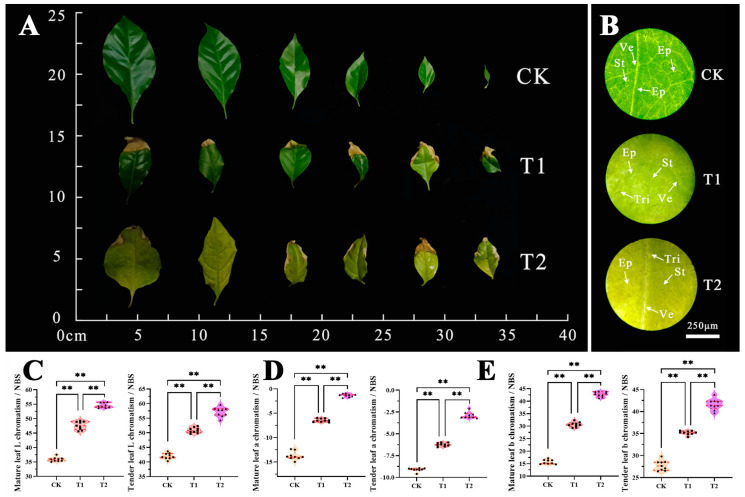
Changes in leaf appearance, anatomical structure, and color differences indicators. (**A**) Morphological changes of mature and tender leaves. (**B**) Leaf surface structure under an optical microscope. Note Ve. vein; Ep. epidermis; St. stoma; Tri. trichomes. Scale bar = 250 μm. (**C**) Leaf L chromatism value. (**D**) Leaf a chromatism value. (**E**) Leaf b chromatism value. Note: ** indicates highly significant differences in the same indicator between different treatments (*p* < 0.01).

The leaf blade is important for photosynthesis, transpiration, and gas exchange. The leaf morphology varied greatly under different fumigation times and treatments, with observable differences between groups (Figure 4A). Compared with the CK, the mature and young leaves of T1-treated *B. glabra* ‘Elizabeth Angus’ had water-stained spots on the leaf edges and a loss of green on the surface. Similarly, the T2-treated *B. glabra* ‘Elizabeth Angus’ leaves experienced more severe damage with a more extensive loss of green color, larger areas of water-stained spots, and prominent damage on leaf edges and surfaces. Microscopic observation further showed the extensive impairment of leaf structure in the T1 and T2 treatment groups. The leaf surface gradually yellowed, and the differentiation and clarity between epidermal cells decreased, while leaf veins and other vascular tissue structures showed inactivation and damage. Additionally, the leaf pattern appeared disorganized, the stomatal openings decreased, and the overall leaf surface exhibited significant damage (Figure 4B).

Under fumigation treatment, the apparent color of *B. glabra* ‘Elizabeth Angus’ leaves gradually became pale, yellowing with a loss in green color. Further analysis of the color changes of *B. glabra* ‘Elizabeth Angus’ leaves confirmed and aligned with the observed traits, validating the occurrence of chlorosis and yellowing in both mature and tender leaves. The results revealed a significant increase in the ‘L’, ‘a’, and ‘b’ values of mature and tender leaves. The positive increase in the ‘L’ value suggested an enhancement in leaf brightness (Figure 4C). A decrease in ‘a’ value indicates a decrease in greenness (Figure 4D). A positive increase in the ‘b’ value indicates an intensification of yellow pigmentation (Figure 4E). Among leaves at different developmental stages, the range of changes in ‘L’, ‘a’, and ‘b’ values is more significant in mature leaves, leading to greater color differences. The above results confirmed that the color difference of *B. glabra* ‘Elizabeth Angus’ leaves increases with increasing concentration and time.

**Figure 5:**
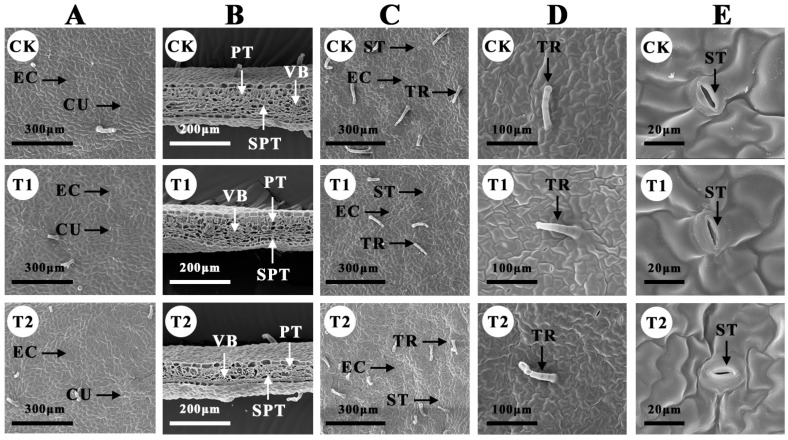
Changes in *B. glabra* ‘Elizabeth Angus’ blade microstructure under different fumigation times of treatments observed by SEM. (**A**) Upper surface, scale bar = 300 μm. (**B**) Crosscutting structure, scale bar = 200 μm. (**C**) Lower surface, scale bar = 300 μm. (**D**) Trichomes, scale bar = 100 μm. (**E**) Stomata, scale bar = 20 μm. Note: EC, epidermal cells. CU, cuticle. VB, vascular bundle. PT, palisade tissue. SPT, spongy tissue. TR, trichomes. ST, stomata.

SEM observation on the microstructure of *B. glabra* ‘Elizabeth Angus’ leaves revealed that the epidermal cells of the leaves in the CK were basically structurally intact after 3 days (Figure 5A). The epidermal cells were tightly arranged, the cell structure was stable, the edge of the cells was clear, and there was no breakage of the cuticle (Figure 5C), whereas the epidermal cells of B. glabra ‘Elizabeth Angus’ treated with intermittent fumigation (T1) showed a certain degree of deformation, and the arrangement of epidermal cells was deformed, and some of them were damaged. The epidermal cells treated with intermittent fumigation (T1) showed some deformation, and the arrangement of the epidermal cells was in certain deformation. Some cells were deformed in size, the cuticle was damaged, and some of them were ruptured, resulting in the exposure of the leaf cells. The epidermal cells treated with continuous fumigation (T2) showed severe deformation, the arrangement of the cells was in severe deformation, and the cuticle was seriously damaged. In addition, the vascular bundles, palisade tissue, and spongy tissues in the CK treatment group were complete and neatly arranged, and the leaf surface was flat (Figure 5B). The palisade tissues of the intermittent fumigation group were basically intact, the spongy tissues were deformed to a certain extent, and the thickness of the leaf blade was thinned in the leaves of the continuous fumigation group (T2). There were large deformations of the palisade and the spongy tissues, and the arrangement of the cells was more disordered, the structure of the vascular bundles was inconspicuous, and the thickness of the leaf blade was seriously reduced. In leaf trichomes and stomata, intermittent fumigation (T1) and continuous fumigation (T2) led to different degrees of trichomes shrinkage and stomatal closure, respectively (Figure 5D,E).

**Figure 6:**
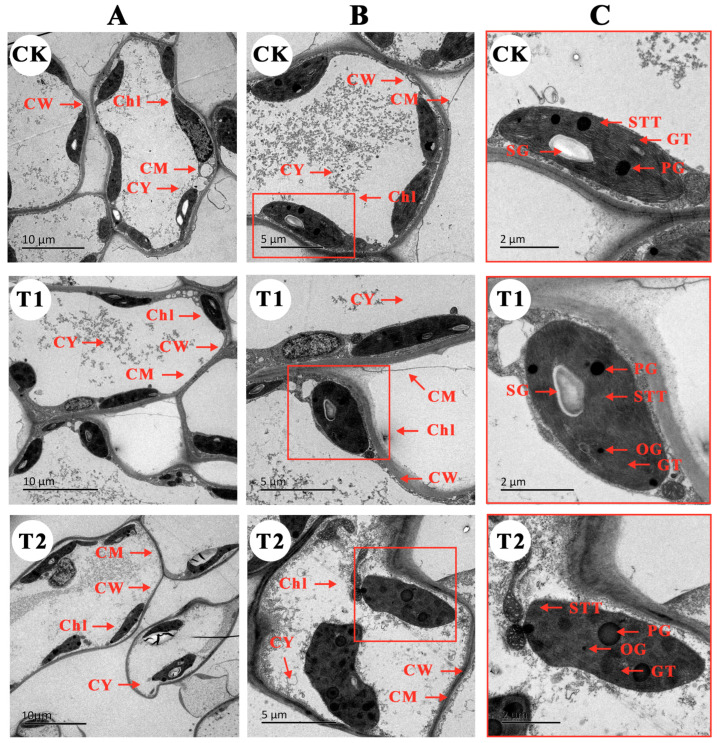
Changes in *B. glabra* ‘Elizabeth Angus’ blade cell ultrastructure under different fumigation times of treatments observed by TEM. (**A**) Overall cellular structure, scale bar = 10 μm. (**B**) Local cellular structure, scale bar = 5 μm. (**C**) Chloroplast, scale bar = 2 μm. Note: CK, control treatment without NO_2_. T1, 4 μL·L^−1^ NO_2_ (8 h/d). T2, EC, 4 μL·L^−1^ NO_2_ (24 h/d). CW, cell wall. CM, cell membrane. CY, cytoplasm. Chl, chloroplast. SG, starch granules. PG, proteinoplast granules. OG, osmiophilic granules. GT, granular thylakoid. STT, stroma thylakoid.

Further observation of the mesophyll cells and chloroplast structure of *B. glabra* ‘Elizabeth Angus’ leaves under different treatments revealed that the leaf cells and chloroplasts of *B. glabra* ‘Elizabeth Angus’ were affected to varying degrees under stress. After 3 days, the CK control group had stable cell structure, clear framework, and intact cytoplasm and cell membrane (Figure 6A). However, fumigation treatment, especially continuous fumigation (T2), caused damage to the stability of the cell structure in the mesophyll cells, resulting in thinning of the cell wall and rupture of partial structures of the cell membrane, leading to leakage of substances in the cytoplasm. Compared with CK control, the chloroplasts of T1 and T2 became irregular in shape, with some organelles detached from the cell wall (Figure 6B). Further observation of chloroplasts revealed that fumigation treatment significantly damaged the chloroplast structure, causing cell swelling and volume increase. The number and volume of starch granules in the chloroplasts decreased, and the number of plastosomes significantly increased. The arrangement of thylakoid grana was loose, the lamellar structure was blurry, and osmiophilic granules significantly increased.

Figure S1 indicated that the length, width, and area of *B. glabra* ‘Elizabeth Angus’ leaves under different concentration treatments did not show significant changes within the treatment group (Figure S1A–C). The growth rate of mature and tender leaves was affected differently based on the observed trend. Among them, mature leaves’ length, width, and area increased to a certain extent under CK treatment, decreased increment under T1 treatment, and slightly decreased under T2 treatment. The changes in leaf length, width, and area of tender leaves were insignificant (Figure S1E–G). With the increase in time and concentration, the quality of mature leaves did not show significant changes under the three treatments (Figure S1D), while the quality of tender leaves showed a significant downward trend with the increase in treatment concentration and time (Figure S1H). Thus, it is evident that the NO_2_ treatment with 4 μL·L^−1^ significantly impacts the quality decline of the tender leaves of *B. glabra* ‘Elizabeth Angus’.

Different from the changes in leaf length and width indicators, the thickness of mature and tender leaves of *B. glabra* ‘Elizabeth Angus’ varied significantly under different concentration treatments (Figure S2A,E). The thickness of mature and tender leaves decreased by 6.377% and 6.763%, respectively, within 72 h (Figure S2B,F). However, the volume of leaves at different stages of development remained relatively stable over 72 h (Figure S2C,G). The specific leaf area index of mature leaves showed a temporal and concentration-dependent pattern, initially increasing and subsequently decreasing (Figure S2D). In particular, the surface area significantly increased to 33.59 m^2^/kg in the T1 treatment after 72 h, compared to only 17.99 m^2^/kg in the T2 treatment (Figure S2H). The specific leaf area of the two treatments was 152.89% and 81.90% of the CK treatment during the same period, respectively. The specific leaf area of tender leaves is generally similar, but the maximum T1 and minimum T2 values at 72 h were 103.76% and 75.88% of CK at the same period, respectively. The minimum specific leaf area of mature and tender leaves is treated with T2 for 72 h.

#### 2.1.5. Changes in Bract Appearance, Anatomical Structure, and Color Differences Indicators

**Figure 7:**
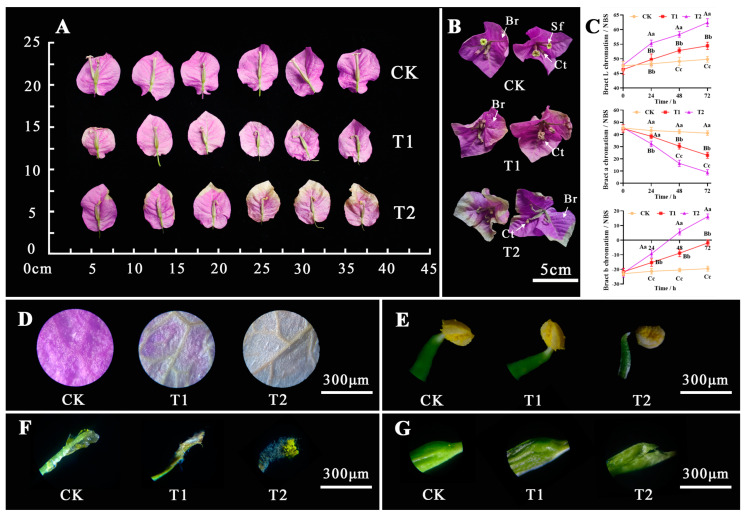
Changes in bract appearance, anatomical structure, and color differences indicators. (**A**) Bract morphology and true flower. (**B**) The degree of starflower opening and the color of bracts. Note: Br. bract (modified leaf); Ct. corolla tube (true flower); Sf. starflower. Scale bar = 5 cm. (**C**) Bract Lab chromatism value. Note: Capital letters (*p* < 0.01) and small letters (*p* < 0.05) indicate significant differences, respectively. (**D**) Surface structure of bracts. (**E**) Stamens. (**F**) Pistils stigma. (**G**) Pistils ovary. Scale bar = 300 μm.

The bracts of *B. glabra* ‘Elizabeth Angus’ are atypical leaves and are the most ornamental of the Bougainvillea. The bracts showed obvious differences under different concentrations of NO_2_ stress treatment. High concentrations of NO_2_ treatment have a negative impact on the normal growth and development of the bracts and flowers. Moreover, the higher the concentration, the faster the color of the bract changes from purple-red to yellow and white (Figure 7A). The overall structure of bracts also tended to be unstable, with a higher degree of wilting, a lower degree of stellate flower opening in the corolla tube, and a more unstable corolla tube structure (Figure 7B).

The color change of bracts is determined by the content and ratio of betacyanins and betaxanthins in the plant body, and adverse conditions can accelerate the decomposition of pigments. The figure illustrates notable variations in the L, a, and b values of bracts between different treatments, which closely resemble the color difference changes observed in the leaves. The L and b values of bracts continue to increase (Figure 7C), while the a value continues to decrease. As the concentration and time increase, *B. glabra* ‘Elizabeth Angus’ bracts gradually turn bright and yellow. Compared with CK, the concentration of betacyanins in the bracts decreases, resulting in a lighter shade of purple-red color. The results demonstrate a positive correlation between the concentration and duration of exposure and the amount of peel-off from the bracts of *B. glabra* ‘Elizabeth Angus’. Moreover, the color expression and morphological structure of the bracts progressively deteriorate as the concentration and duration of exposure increase.

Microscopic observation further revealed that the bracts of *B. glabra* ‘Elizabeth Angus’ changes were similar to those of the leaves. The bract surface gradually transitions from purple to a lighter shade (Figure 7D). The continuous decomposition of betacyanin pigment leads to noticeable changes in the conducting tissues, resulting in a decrease in the overall thickness of the bracts. In the actual floral organs, except for the structural deformation of the pistil stigma T1 and T2 treatments, no significant changes were found in the morphology of the stamens and ovaries (Figure 7E–G).

### 2.2. Changes in Tissue Moisture Content and Pigment Content

There is a close relationship between plant metabolism and water content, and stress can induce alterations in the water content of plants. Different fumigation times of NO_2_ treatment resulted in a general decrease in the water content of various organs of *B. glabra* ‘Elizabeth Angus’ (Table 1). Unlike CK, no significant difference in water content was observed in the bract organs of the T1 stem and leaf except for the root. However, the difference in water content between different organs of T2 was more significant, especially the root water content. The average values decreased by 6.78 and 3.57 percentage points compared to CK and T1, respectively. Among all organs, the relative decrease in root water content before and after treatment was the highest. However, the leaf water content exhibited the most significant absolute decrease before and after treatment compared to other organs, with only 78.276% under T2 treatment. There is still a significant decrease in the water content of the entire plant in conditions T2, CK, and T1.

Plants’ contents and proportion of pigments are important indicators for determining their apparent traits and measuring their photosynthetic potential. As shown in Table 2, different times treatments significantly differ in the photosynthetic pigment content of different *B. glabra* ‘Elizabeth Angus’ organs. Among the total chlorophyll (Chl) and carotenoid (Car) indicators, the changes in leaves are opposite to the increase in stem and bract content. The content of total chlorophyll and carotenoids in leaves decreased significantly by 51.3% and 73.74% compared to the CK, respectively, but still significantly exceeded the chlorophyll content in stems and bracts. The changes in the content of different chlorophyll a (Chl-a) and chlorophyll b (Chl-b) organs are consistent with the total chlorophyll. Except for a significant decrease in leaves, both the stem and bracts show varying degrees of increase. The above findings suggest a reduction in leaf pigmentation and increased stem and bract pigmentation in *B. glabra* ‘Elizabeth Angus’ before and after treatment.

### 2.3. Changes in Chlorophyll Fluorescence Parameters

Chlorophyll fluorescence is a more accurate indicator of chlorophyll concentration and photosynthetic capacity in leaves under different environments. Table 3 shows the overall trend of chlorophyll fluorescence parameters and chlorophyll content changes in *B. glabra* ‘Elizabeth Angus’ are similar. The relative fluorescence parameters of *Fo*, *Fv*, and *Vi* showed significant differences under different treatments, while the differences between different treatment groups are not significant compared to *Fm* and *Vj.* Additionally, among the optical system parameters, except for *Φ*(*Do*) (the maximum quantum yield of non-photochemical quenching), all other indicators showed significant differences between treatment groups. Particularly, *Φ*(*Eo*) and *ψ*(*Eo*) of T2 decreased by 72.59% and 66.63% compared to CK, respectively. There was no significant difference between the *ABS*/*RC*, *ETo*/*RC*, and *REo*/*RC* of unit reaction center parameters, while the leaf performance index *PI* (*ABS*) showed significant differences among the treatments.

### 2.4. Changes in Photosynthetic Parameters

Photosynthetic parameters provide a more accurate assessment of the photosynthetic capacity of plants during specific growth stages or in different living environments. There are significant differences in the photosynthetic system parameters of *B. glabra* ‘Elizabeth Angus’ leaves at different concentrations (Table 4). The net photosynthetic rate (Pn) showed significant differences under treatments, with T1 and T2 treatments decreasing by 31.25% and 55.87% compared to CK, respectively. The difference in intercellular CO_2_ concentration between treatment and CK control was extremely significant, while there was no significant difference between T1 and T2. There are at least two groups with insignificant differences in Gs, Tr, VPD, and WUE indicators between the treatment group and the CK control group. The above results indicated that NO_2_ stress treatment significantly affects Pn and Ci, while the impact on other photosynthetic indicators is not statistically significant.

### 2.5. Comprehensive Analysis of Response Differences and Evaluation of Membership Functions

**Figure 8:**
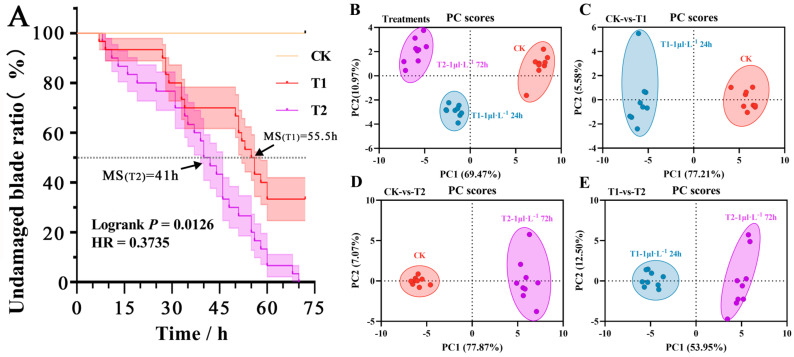
Leaf damage situation correlation heatmap of significant differences in *B. glabra* ‘Elizabeth Angus’ indicators under different fumigation times treatments. (**A**) Change of undamaged blade ratio. MS. median survival; HR. hazard ratio. (**B**) Principal component analysis (PCA) of response indicators under different treatments. (**C**) PCA scatter scores for CK and T1. (**D**) PCA scatter scores for CK and T2. (**E**) PCA scatter scores for T1 and T2.

Under different concentration treatments, there are certain differences in the survival status of the leaves of *B. glabra* ‘Elizabeth Angus’ seedlings. The experiment used the damaged leaf area reaching half of the total area of a single plant as the half-lethal dose of the leaves and compared and analyzed the damage of the leaves over time under different treatments. The results demonstrated that the time for *B. glabra* ‘Elizabeth Angus’ leaves to reach the half-lethal dose was significantly accelerated in the various times treatments compared to the control group. The median survival period of the leaves of *B. glabra* ‘Elizabeth Angus’ seedlings under T1 and T2 treatments were 41 h and 55.5 h, respectively (Figure 8A). After 72 h of stress treatment, the respective counts of cyclamen plants that did not reach the half-lethal dose were 10 and 0. The results of the significance analysis indicate that *p* = 0.0126 and the hazard ratio is 0.3735 < 1. These findings suggest that the leaf damage in the T1 treatment is slightly superior to that in the T2 treatment.

Meanwhile, the comprehensive effects of NO_2_ treatment at different concentrations and durations on the morphology and physiology of *B. glabra* ‘Elizabeth Angus’ seedlings were evaluated through correlation analysis and principal component analysis on various indicators of the CK control and the T1 and T2 treatment groups. Based on the significance of data differences between treatment groups (*p* < 0.05) and the premise of principal component analysis, the requirements of a positive definite matrix and KMO test greater than 0.6 were met for each indicator. The 72 h mean values of the 50 measured indicators in the experiment were used as the baseline data (Figure 8B). To remove dimensional differences between indicators, the data for principal component analysis were standardized in this study. The KMO test and Barrett spherical test were satisfied (KMO = 0.701; Sig = 0.00), the initial characteristic value was greater than 1, and the cumulative contribution rate exceeded 90%. As a result, a hierarchical index component factor load matrix diagram and 23 principal component screening indicators with their parameters were obtained (Table 5). The results of the two treatment groups showed a significant difference in sample population between the two groups, while the difference in indicators under the same treatment was minimal (Figure 8C–E).

**Figure 9:**
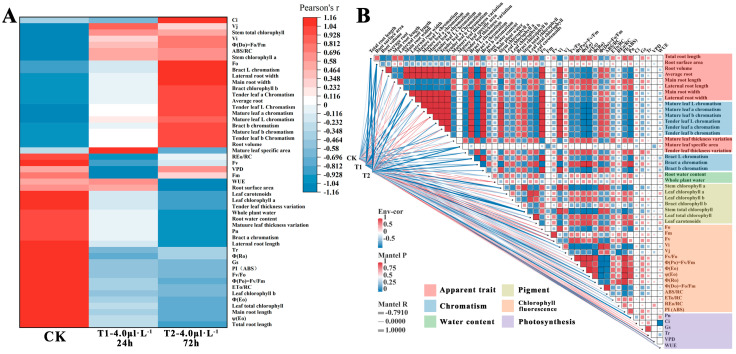
Cluster analysis of the correlation between different indicators and treatments. (**A**) Correlation between indicators analyzed by Pearson index method. (**B**) Clustering of indicators and treatment groups based on correlation. Note: Red represents a positive correlation between indicators. Blue represents the negative correlation between indicators. The depth of color is directly proportional to the absolute value of the correlation coefficient.

**Figure 10:**
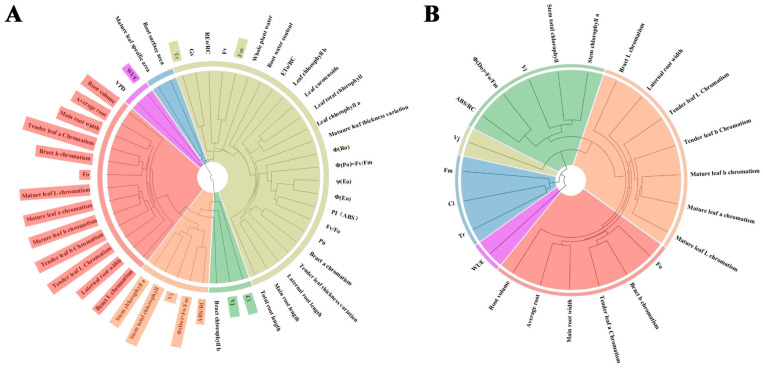
Cluster analysis of indicator systems before and after principal component screening. (**A**) Systematic clustering analysis of the original 50 indicators before PCA. Note: Colored indicators represent indicators selected for principal component analysis. (**B**) Systematic clustering analysis of 23 indicators selected by PCA.

In addition, a notable gradient is observed in the correlation clustering among all indicators and between processing and indicators. The changes in all indicators between the three treatments can be roughly divided into three categories (Figure 9A). The first category of indicators has a continuous increase in correlation *p*-values between CK and T2, representing indicators such as *Fo*, bract L chromatism, main root width, etc. The second category of indicators shows unstable correlation *p*-values between CK and T2, representing indicators such as *REo/RC*, *VPD*, *Fv*, *Fm*, etc. The third category of indicators shows a continuous decrease in correlation *p*-values between CK and T2, representing indicators such as Pn, *Fv/Fo*, etc. The correlation between different indicators shows clustering characteristics (Table S1). For example, there is a strong correlation between indicators mainly related to root and leaf morphology, and different chlorophyll fluorescence parameters also show a strong correlation (Figure 9B). The clustering between indicators before and after PCA screening also indirectly confirms the clustering of strongly correlated indicators (Figure 10A). Based on the clustering between treatments and indicators, the three categories of indicators are further divided into six categories. The weakly correlated indicators in the first category are further divided into other groups, such as *Vi* and Ci. Similarly, the second indicator group was further divided, but the third indicator displayed minimal changes and exhibited insignificant variation in correlation with the treatments. Similar indicators were further screened in the PCA process (Figure 10B).

In the PCA analysis, based on Formulas (1) and (2), the results (Table 5) demonstrated that the feature values of the three principal components after λ screening are all greater than 1, with a cumulative contribution rate of 94.995% (>90%). The contribution rate of the first principal component is 76.273%, representing 76.273% information of 23 indicators. The first principal component, PC1 (θ), has a high loading capacity for the relevant parameters of the root as well as the Lab values of the leaves and bracts. Similarly, the second principal component, PC2 (θ), shows that the absolute load values of *Fm*, Ci, Tr, and WUE indicators in the middle exceed the 0.5 thresholds. In the third principal component, PC3 (θ), only the absolute loadings values for the *Fm*, Tr, and WUE indicators exceed 0.4. The ranking of comprehensive scores for the three principal components is as follows: CK (−4.135) < T1 (0.317) < T2 (3.818). The above results indicate that the stress of NO_2_ primarily affects *B. glabra* ‘Elizabeth Angus’ in changes in organ morphology and color parameters, as well as a decrease in leaf photosynthesis. Notably, CK obtains the lowest score in the comprehensive morphology index (Table 6).

Principal component screening is conducted on 23 indicators under different treatments, and the analysis is based on fuzzy mathematical membership functions. The comprehensive scores of each indicator are determined using Formulas (3) and (4), followed by a weighted average calculation. The results are shown in Table 7. The CK control exhibited high scores, close to the maximum value of 1, in indicators negatively correlated with treatment concentration and time. Some photosynthetic indicators, such as Ci, Tr, and WUE, did not show significant differences compared to the treatment group. The scores of various indicators in the T1 treatment group are mainly concentrated at 0.5. The T2 treatment had the lowest scores for various indicators, with most indicators scoring less than 0.2. The comprehensive scores and apparent traits of each treatment group showed similar differences, with CK (0.8657) > T1 (0.4715) > T2 (0.1982). The results indicate that T2 treatment induces the most significant damage to plants under stress.

## 3. Discussion

### 3.1. NO_2_ Stress Alters Apparent Traits, Morphological, and Anatomical Structures

When plants are exposed to stress, the most direct manifestation is the response of apparent traits. However, plant responses are influenced by various internal and external factors, including the type and intensity of stress and plant characteristics. *B. glabra* ‘Elizabeth Angus’ responds greatly under drought, saline soil, and different gas pollutants [24,28]. In this study, different organs showed certain morphological differences, with similarities observed in the changes of roots, stems, and petioles before and after. Overall, these materials exhibit color fading, structural gaps, and a reduction in the thickness and strength of the layers, consequently impeding or halting physiological activities. Furthermore, both the transportation of goods and assimilation capabilities have been impaired to different extents. Prolonged exposure to high concentrations of NO_2_ significantly degraded chlorophyll in the leaves [29,30], leading to severe bract discoloration and the development of spots and faded water stains on leaf edges and middle sections. Anatomical observations show that the stratum corneum beneath the leaf’s epidermis has been severely damaged, including upper epidermal cells and sections of the barrier tissue that have also experienced mechanical injuries. We hypothesize that this phenomenon may result from leaf transpiration or guttation [31], in which nitric acid is generated from NO_2_ and H_2_O, leading to the deterioration of leaf surface structure. Moreover, the leaf epidermis also demonstrates similar damage to vascular tissues induced by stress, protoplast rupture, decreased stability of intercellular structures, and impaired stomatal performance. Mature and tender leaves have poor resistance responses to stress. The environmental changes have influenced the abundance of authentic flowers in *B. glabra* ‘Elizabeth Angus’. Compared with previous studies, it is speculated that NO_2_ may impact a specific transcription factor’s regulatory activity, resulting in trait variations [32,33]. However, no significant changes were found in the morphology and function of real flower organs in anatomical structure, which differs from the expected results of previous cold resistance experiments where flowers were initially damaged. Experimental evidence has shown that NO_2_ has the potential to stimulate sexual reproduction in tomatoes [34]. Nevertheless, additional research is required to examine the pollen viability and sexual reproduction of *B. glabra* ‘Elizabeth Angus’ [35,36,37]. The length, thickness, and crown width of stems do not exhibit significant changes before and after stress due to the correlation between processing time and concentration and the comparatively slower growth rate of stems than leaf changes.

In this study, the majority of data collected focused on leaf indicators. However, no significant alterations were observed in leaf volume, length, width, area, or mature leaf quality. The slow or even negative increase in leaf thickness and quality in mature and tender leaves indicates that NO_2_ stress weakens leaf growth functional traits. Leaves, similar to other stresses, undergo self-adjustment mechanisms to effectively adapt to environmental changes [38]. The decrease in leaf thickness is a distinct characteristic resulting from exposure to NO_2_ stress, specifically caused by the formation of nitric acid through the interaction between NO_2_ and plant guttation. The above results indicate that the leaves, roots, and bracts of *B. glabra* ‘Elizabeth Angus’ are more sensitive organs to NO_2_ stress. Moreover, the biochemical characteristics of the leaf surface can be utilized as indicative markers. The reduction in growth rate and the decrease in the length of internodes in real flowers and stems during periods of stress can be considered a self-protective mechanism. Considering other morphological indicators of various organs, we observed significant changes in root length, thickness, surface area, and leaf-bract color difference following stress. Meanwhile, we have found significant differences between various treatments [39]. Based on the aforementioned observations, these indicators can be considered sensitive for distinguishing between treatments.

### 3.2. The Response Mechanism of Water and Photosynthetic Physiology to NO_2_

Water is an essential requirement for the growth and development of plants. Plants’ compositions and physiological activities depend on water’s presence. Environmental conditions strongly influence the availability of water in plant tissues. Previous studies have demonstrated that exposure to SO_2_, formaldehyde, and O_3_ stresses leads to a decline in both free water and bound water in plants [25,40], with formaldehyde showing the most significant decrease [38]. The rate of water decline in the *B. glabra* ‘Elizabeth Angus’ under NO_2_ stress is moderate among the above pollution gases. The results revealed variations in water content among different organs of *B. glabra* ‘Elizabeth Angus’ before and after treatment. Generally, the root experiences the largest percentage decrease and serves as the primary organ responsible for water absorption. In the experiment, while NO_2_ did not directly enter the soil, the reduction in stem and leaf water content could enhance root water transport, thus indirectly impacting notable alterations in root apparent characteristics. However, the water content of the leaves decreased to less than 80% after stress, which is consistent with the effect observed from previous pollutants [25].

In addition, there is a strong consistency in indicators related to plant photosynthesis, such as pigment content, fluorescence kinetics parameters, and photosynthetic indicators [41,42]. The alteration of pigment proportions in plants is an adaptive strategy for adapting to the environment. T1 treatment resulted in a certain increase in bracts and stems, while the chlorophyll content in the leaves shows a significant decrease, which is consistent with the results of apparent traits. The leaves displayed a lighter color, with disintegrated chlorophyll and an increased proportion of chlorophyll a/b, indicating that the photosynthetic organs of *B. glabra* ‘Elizabeth Angus’ were damaged, leading to a reduced capacity to utilize weak light and synthesize assimilation products. Notably, certain situations in the T1 treatment group reached their maximum values, with an observed increasing trend in the stem and bracts. This finding indirectly confirms that *B. glabra* ‘Elizabeth Angus’ has a certain self-regulation ability within the tolerance threshold range and tends to exhibit a passive mode of adaptation in response to moderate to high concentrations [10].

Chlorophyll fluorescence and photosynthetic parameters can effectively reflect real-time photosynthesis and potential capacity in plants. Previous studies have shown that exposure to NO_2_ stress at concentrations of 12 mg·m^−3^ and 4 μL·L^−1^ resulted in an increase in *Fo* and a subsequent reduction in *Fv*/*Fm* of photosystem II in *Bougainvillea spectabilis* and *Carpinus turczaninowii*, respectively [7,18]. This experiment observed a similar effect after 72 h of treatment with NO_2_ at a concentration of 4 μL·L^−1^. Generally, higher initial fluorescence Fo indicates stronger stress and adversity for plants, whereas *Fm* and *Fv* exhibit the opposite trend. The changes in the three indicators in this study were similar and consistent with the initial predictions [43]. *Vi* and *Vj* represent the relative variable fluorescence intensity at points I and J, respectively, which are significant indicators of the OJIP curve. The varying degrees of increase in these two indicators at T1 and T2 suggest that the fluorescence curve of *B. glabra* ‘Elizabeth Angus’ has undergone adjustments to adapt to the environment. The increase in Vj during T1 treatment is less than *Vi*, indicating that the transmission of electrons from Q_A_ to Q_B_ is blocked. The accumulation of electrons in Q_A_ indicates that the damaged site is within Q_A_ [44].

Optical system parameters *Φ* (*Po*), Fv/Fo, and *Φ* (*Do*) represent the maximum quantum yield of photosystem II, the potential quantum yield, and the maximum quantum yield of non-photochemical quenching, respectively. The significant changes between different treatments indicate that the PSII photosystem is disrupted under stress conditions in the triangular plum. The absorption of light energy by the unit reaction center *ABS*/*RC* has increased, but there has been a decrease in *ETo*/*RC* for electron transfer energy and *REo*/*RC* for light energy transferred to PSI, which suggests that the absorbed energy by the reaction center has not been effectively transmitted to the receptor site for physiological reactions. The *PI*_(*ABS*)_ indicates the overall state of the plant’s photosynthetic structure and provides a comprehensive assessment of the absorption, capture, and transportation of PSII reaction center complex photosynthesis. The decreased *PI*_(*ABS*)_ in this study indicates a decline in the photosynthetic performance of the PSII system. Moreover, the reduction in both the net photosynthetic rate (Pn) and the transpiration rate (Tr) observed in the photometric indices aligns with the anticipated outcomes [45]. However, the later increase in intercellular concentration indicates that the decline in Pn is not solely attributed to stomatal factors, and there is a strong association between the increase in VPD and the decrease in WUE. Indeed, treatment with concentrations higher than 1.0 μL·L^−1^ typically reduces photosynthetic rate among most plant species [41].

### 3.3. Systematic Evaluation and Analysis of Response Mechanisms

In this study, a mass flow controller and cloud platform were employed to monitor data and effectively maintain the gas concentration in the laboratory at a range of 4.0 μL·L^−1^ ± 0.1 μL·L^−1^. In contrast to formaldehyde and dust, NO_2_ is a gas with dualistic properties. On one hand, fumigating plants with NO_2_ does not inhibit or stimulate plant growth in a short period. This phenomenon is known as the “activation effect”, characterized by NO_2_ compensation points [46]. For example, in *Arabidopsis thaliana* at low concentrations (<1 μL·L^−1^), the leaf area exhibits a notable increase [47].

On the other hand, NO_2_, as a signaling molecule, also has adverse effects on plant metabolism. Therefore, research on the semi-lethal concentration of NO^2^ has demonstrated that plants should not endure it for more than 48 h when exposed to a concentration of 8 mg·m^−3^ [48]. This study demonstrated that subjecting plants to continuous, short-term, high-concentration treatment for 72 h led to irreversible damage. Interestingly, in the subsequent recovery experiments, the *B. glabra* ‘Elizabeth Angus’ treated with T1 could recover by replacing damaged, mature leaves with tender leaves, while there was no similar renewal phenomenon under T2 treatment.

The Kaplan-Meier (K-M) curve is commonly used to analyze changes in overall survival status between the treatment and control groups in the presence of a specific toxic effect. The statistical results of the proportion of undamaged leaves in this study showed that the expected gradient (CK > T1 > T2) was observed. During the 72 h of continuous pollution, damaged leaves had a median survival time of 41 h. Furthermore, the HR (hazard ratio) for the intermittent fumigation was only 0.3735, significantly lower than the mortality rate observed with continuous fumigation (T2). The 23 indicators selected in PCA are more effective in representing the main influencing factors and their respective weights. Indicators showing significant differences among different groups are more frequently observed in the final load matrix. Among them, root, leaf color, bract color, and chlorophyll fluorescence indicators can be the primary indicators to assess the resistance and adaptability of *B. glabra* ‘Elizabeth Angus’. This finding demonstrates similarities between the composite gas experimental studies conducted on highways in India and Egypt [49,50]. The subsequent membership function scores further confirmed the consistency of the phenotypic traits, specifically CK > T1 > T2. Further investigation is necessary to examine the specific alterations in metabolites and the corresponding regulation of transcription factors during stress in this study. Subsequent research can focus on enhancing the resistance of *B. glabra* ‘Elizabeth Angus’ through exogenous active substances [51,52,53].

In summary, this study conducted a 72 h NO_2_ airtight fumigation test on the resistant variety *B. glabra* ‘Elizabeth Angus’ using three treatments. The results indicated that NO_2_ concentration of 4 μL·L^−1^ caused significant damage to the PSII photosynthetic system and apparent traits of *B. glabra* ‘Elizabeth Angus’, which resulted in the inactivation of the root system and reduced its water absorption capacity by harming the root structure. Decreasing the concentration of photosynthetic pigments and water in leaves and bracts leads to a lighter and yellowish color and the erosion of vascular tissue and epidermis by nitric acid. In addition, the photosynthetic capacity of the PSII photosynthetic system within the leaves was continuously disrupted and declined. There were no significant changes observed in the stem and floral organs. Additionally, the opening of the real flower corolla tube was found to be closed under NO_2_ induction. K-M survival curve analysis was conducted continuously for 72 h. The median survival time of 4 μL·L^−1^ fumigation was 41 h. Root and leaf Lab color differences, chlorophyll fluorescence, and Pn can be utilized as stress indicators.

## 4. Materials and Methods

### 4.1. Materials

**Figure 11:**
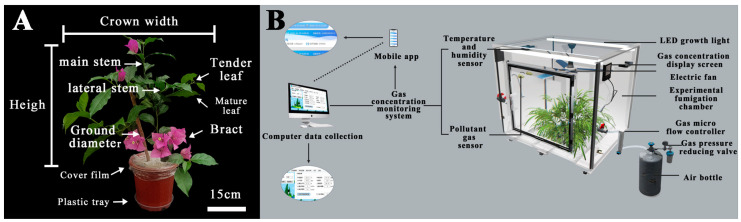
Experimental plant materials and fumigation test equipment. (**A**) *B. glabra* ‘Elizabeth Angus’ (2-year-old cutting seedling). Scale bar = 15 cm. (**B**) Schematic diagram of NO_2_ fumigation test device.

Two-year-old seedlings of *Bougainvillea glabra* ‘Elizabeth Angus’ were purchased as cuttings from the Xiaxi Flower and Wood Market in Changzhou in September 2022 (Figure 11A). The plants were cultivated with planting soil (loam soil: peat: perlite = 1:1:1) in the greenhouse seedbed of the Garden Experimental Teaching Demonstration Center of Nanjing Forestry University (32°09′86′′ N, 118°81′68′′ E) and conducted unified water and fertilizer management. Winter insulation management was implemented between December 2022 and March 2023, with regular management practices applied during other periods. To maintain soil moisture in the pot during the management period, a plastic water tray was positioned beneath the potting soil (Figure 11A). Hoagland’s nutrient solution is sprayed every 15 days to supplement nutrients. During the flowering period in April, additional fertilizations are conducted weekly to ensure consistent flower characteristics and provide an adequate supply of nutrients.

### 4.2. NO_2_ Fumigation Treatment and Samples Collection

In early April 2023, a total of 90 potted seedlings with flower buds (Figure 11A) and plant height of 45 cm ± 5 cm were selected, and then they were transferred to a fully automated artificial fumigation box (120 cm × 120 cm × 90 cm) for one month to acclimate to the indoor environment beforehand. Three days before the formal commencement of the experiment, we applied a covering film around the root neck and below the *B. glabra* ‘Elizabeth Angus’ plant to prevent gas from entering the soil. A tray was also used to prevent water from draining out and ensure adequate water retention in the pot (Figure 11A).

The experiment was designed as a single-factor, completely random variable experiment, and the experimental group maintained relatively consistent environmental factors except for the treatment time. Based on previous concentration studies and pre-experimental results, two treatments were established: T1: 4 μL·L^−1^ NO_2_, daily ventilation time from 09:00 to 17:00, a total of 8 h (4 μL·L^−1^ NO_2_ + 8 h/d), clean air for other times; T2: 4 μL·L^−1^ NO_2_ for all days inside the box (4 μL·L^−1^ NO_2_ + 24 h/d). At the same time, set the control group CK: clean air (0 μL·L^−1^) in the box all day long (0 μL·L^−1^ NO_2_ + 24 h/d). Ten plants were used per treatment, with a total of 3 replicates in a treatment. For the experiment, the test gas was sourced from Nanjing Changyuan Industrial Gas Co., Ltd. (Nanjing, China), with a gas concentration of 4 μL·L^−1^_._ The gas concentration in the fumigation box was controlled by the ALICAT MC mass flow controller (1 sccm = 1 mL·min^−1^) with a concentration of 4.0 μL·L^−1^ ± 0.2 μL·L^−1^. In short, each box is taken for fumigation for approximately 315 s (4.0 μL·L^−1^ × 1296 L = 5.184 mL, 5.184 mL × 1 sccm = 5.184 min ≈ 315 s) and subsequent gas replenishment. Detector and real-time concentration data cloud platform were provided by Weihai Jingxun Tongtong Electronic Technology Co., Ltd. (Weihai, China) (Figure 11B). The fumigation test started on 1 May 2023, spanned 3 days, and lasted 72 h.

### 4.3. Measurement Indicators and Methods

#### 4.3.1. Morphological Indicators and Anatomical Structure

Morphological indicators of different organs of *B. glabra* ‘Elizabeth Angus’ were measured at 08:00 every day (Figure 11A), and the color measurements were conducted using a 3 nh spectrophotometer equipped with a built-in D65 standard light source. An 8 mm window diameter and an observation angle of 10° were employed. The color Lab measured the roots, leaves, and bracts of each plant as the top 1–3 tender leaves (Figure 11A), the top 3–5 fully functional leaves (Figure 11A), and the middle part of the top bracts. Three plants per group were randomly measured, and each plant was repeated three times to obtain the average value.

The total root length, root surface area, average root diameter, main lateral root length, and roughness were measured using the Wanshen LA-S plant root analyzer (Guangzhou Wanshen Testing Technology Co. Ltd., Guangzhou, China). The length and thickness of the main and lateral stems, ground diameter (diameter 5 cm above the base of the plant), plant height (height from base to top bud) (Figure 11A), and crown width (Figure 11A) were measured using vernier calipers and steel rulers. The length, width, and area of the leaves were measured using the Wanshen LA-S series plant image analyzer, the mass of the leaves was measured using the Mettlertoledo 1/10,000 balance, and the volume of the root and leaf were measured using the drainage method. 

The root system, branches, petiole transverse structure, leaf surface structure, bract surface structure, and floral organs (stamens, stigma, ovary) were examined by immersing the sampled sections in water. Temporary slides were observed under an optical microscope (Olympus CX33, Tokyo, Japan), and photographs were collected. The specific process of leaf microstructure sampling and observation is as follows: the test plant leaf was bladed to take a number of small pieces of 5 mm × 5 mm, quickly submerged in 2.5% glutaraldehyde solution, then fixed with osmium acid at 4 °C for 24 h, rinsed three times with phosphate buffer (pH = 7.2), and sprayed after the dehydration process had reached the critical point, and a scanning electron microscope (SEM) (Regulus 8100 Cold Field Emission SEM, Hitachi, Japan) was used to observe and photograph the front and back surfaces and sections of *B. glabra* ‘Elizabeth Angus’ leaves.

The process of observing the ultrastructure of leaf organelles was basically similar, and a number of small pieces of 2 mm × 2 mm leaves were cut with a knife and quickly submerged in 2.5% glutaraldehyde solution, rinsed three times with phosphate-buffered solution (pH = 7.2), and dehydrated and then embedded in 100% embedding agent. After immersion for 24 h, the embedded sections were stained, then observed and photographed by transmission electron microscopy (TEM) (JEM−1400).

#### 4.3.2. Tissue Moisture Content and Pigment Content

The sampling sites for measuring water and pigment content in various organs are consistent. The roots were the cuttings’ primary roots, the primary lateral stem on the cutting main stem, the fully functional leaves located from the top of the branches to the 3rd to 5th segments below, and the bracts were the mature bracts. The procedure for determining the water content of various organs and tissues under different treatments is as follows: Once fumigation is finished, any surface moisture and dust on each organ are eliminated using dry paper, and then the fresh weight (FW) is measured using an electronic balance. Then, the fresh weight (FW) is subjected to thermal treatment at 105 °C for 0.5 h in an oven, followed by drying to a constant weight at 65 °C. The dry weight (DW) is measured, and the relative water content ((RWC) = (FW − DW)/FW) and specific leaf area (SLA) are calculated. The method for determining the content of chlorophyll is as follows: For each organ sample, the surface is carefully rinsed with deionized water. Then, 0.2 g of the sample is weighed and soaked in 5 mL of a 95% ethanol solution under dark conditions until the sample completely changes to white. The blank control was prepared using 95% ethanol. Enzyme-linked immunosorbent assay measured the optical density (OD) values at 665, 649, and 470 nm for various sample extracts. Each sample was repeated three times to obtain the average value. The content of chlorophyll a, chlorophyll b, total chlorophyll, and carotenoids in the samples was then calculated using the corresponding absorbance value formula sequentially [3].

#### 4.3.3. Chlorophyll Fluorescence Parameters

The measured chlorophyll fluorescence parameters include initial fluorescence (*Fo*), maximum fluorescence (*Fm*), variable fluorescence (*Fv*), the point I relative variable fluorescence (*Vi*), point J relative variable fluorescence (*Vj*), photosystem II maximum quantum yield (*Φ*(*Po*) *= Fv*/*Fm*) and the other form (*Fv*/*Fo*), electron transfer quantum yield (*Φ*(*Eo*)), the capture of light energy for energy ratio for electron transfer (*ψ*(*Eo*)), electron reduction efficiency at the PSI acceptor side end (*Φ*(*Ro*)), maximum quantum yield for non-photochemical burst (*Φ*(*Do*) *= Fo*/*Fm*), absorbed energy (*ABS*/*RC*), energy used for electron transfer (*ETo*/*RC*), light energy for transfer to PSI (*REo*/*RC*), and performance index based on absorbed light energy (*PI*_(*ABS*)_). Measurements were conducted daily from 08:00 to 09:00 a.m. The instrument used for measurement was a HandyPEA high-speed continuous excitation fluorometer with a light intensity of 3000 μmol·m^2^·s^−1^, a light source of 630 nm red light, and a recording interval of 2 s. The leaves were dark-adapted using leaf clips for 30 min before measurement. Each plant was selected from the top to the bottom of 4–6 nodes of the main branch with the same sunny leaf, and each functional leaf measurement was repeated 3 times, and the average value was obtained.

#### 4.3.4. Photosynthetic Parameters

Photosynthesis-related parameters (net photosynthetic rate (Pn), intercellular CO_2_ concentration (Ci), stomatal conductance (GS), transpiration rate (Tr), water utilization (WUE), and water vapor pressure deficit (VPD)) were measured by a CIRAS-3 photosynthesizer. The photosynthetic indexes were measured between 9:00 a.m. and 11:00 a.m. on a clear and windless morning. Three sunny mature leaves from the top to the bottom 4–6 nodes of the main branch were selected from each plant for measurement. Each measurement was repeated three times. The measurements were conducted under consistent conditions, including a leaf chamber temperature of 25 °C, a leaf chamber relative humidity of 75%, and CO_2_ concentration (380–420 μmol·mol^−1^). The photosynthetically active radiation parameter (PAR) was also set to 1000 µmol·m^−2^·s^−1^.

#### 4.3.5. Statistical Data Analysis and Processing

Experimental data were processed using Excel 2016. Charts were generated using GraphPad Prism 9 and Origin software. Statistical analysis was conducted using SPSS 25.0 software for One-Way-ANOVA, and the data was analyzed using Tukey Multiple Comparisons for inter-group significance (*p* < 0.01, *p* < 0.05). All data were the mean ± standard error (Mean ± SE) of 3 replicates.

In principal component analysis, the data are pre-standardized using SPSS, and the Fn and F values for different concentration treatments are calculated using the cumulative contribution rate weights and eigenvalues of the principal components.

Principal component analysis and the membership function were referenced separately, and the key formulas were provided below:

(1) $$F_{i}=wi_{1}X_{1}+wi_{2}X_{2}+\ldots +w_{\mathrm{in}}X_{n},\;w_{\mathrm{ij}}=\theta_{j}/\sqrt{\lambda }i$$

where w_ij_ is the weight of each variable in the principal component, θ_j_ is the corresponding variable coefficients in the component matrix, and $\sqrt{\lambda }i$ is the root value of the eigenvalues corresponding to the i-th principal component.

F = α_1_F_1_ + α_2_F_2_ + … + α_n_F_n_ (2)

where αi represents the percentage of the variance of the i-th principal component.

X (u _positive correlation_) = (X − X_min_)/(X_max_ − X_min_) (3)

X (u _negative correlation_) = 1 − [(X −X_min_)/(X_max_ − X_min_)] (4)

where X represents the measured value of a certain indicator, and X_max_ and X_min_, respectively, represent the minimum and maximum values in a certain indicator [54].

## 5. Conclusions

To investigate the damage mechanism of NO_2_ on *B. glabra* plants and systematically evaluate their resistance, materials with higher resistance in this genus were selected. In this study, the effects of different durations of fumigation with NO_2_ fumigation at a concentration of 4 μL·L^−1^ on the apparent traits, morphology, anatomical structure, water physiology, photosynthetic pigment content, chlorophyll fluorescence, and photosynthetic physiology of *B. glabra* ‘Elizabeth Angus’ were investigated. After evaluating the results under different treatments through principal component analysis, membership function analysis, and K-M survival curve analysis, we conclude that exposure to a high concentration of NO_2_ at 4 μL·L^−1^ for 72 h led to weakened root function, diminished color in the leaves and bracts, reduced chlorophyll content in the stems, and damage to the PSII photosystem, and a decrease in Pn, Tr, and WUE in photosynthetic physiology. During the K-M survival time analysis, the median survival times of the undamaged leaves were determined to be 41 h and 55 h for the 8 h/day and 24 h/day fumigation treatments, respectively. However, the 8 h/day treatment seedings recovered to a certain extent by forming new tender leaves during subsequent regeneration. Prolonged exposure to 4 μL·L^−1^ of NO_2_ can result in irreversible damage to *B. glabra* ‘Elizabeth Angus’, primarily characterized by decreased organ morphological parameters and reduced leaf photosynthesis. Root volume, average root diameter, leaf Lab color difference indicators, chlorophyll fluorescence parameters, net photosynthetic rate, and fumigation time are closely related and can be used as crucial evaluation indicators for the resistance of *B. glabra* ‘Elizabeth Angus’ to NO_2_ stress. This study conducted a comprehensive analysis and evaluation of the morphological characteristics and physiological responses of *B. glabra* ‘Elizabeth Angus’ under a resistance of 4 μL·L^−1^, thus revealing the response mechanism of *B. glabra* ‘Elizabeth Angus’ under medium to high concentrations of NO_2_ stress. The results of this study are conducive to expanding the field of plant environmental ecotoxicology and providing a theoretical basis for the targeted improvement of garden plant resistance and the application of plant NO_2_ reduction.

## Figures and Tables

**Table 1:** Changes in water content of different organs of *B. glabra* ‘Elizabeth Angus’ under different treatments.

Treatment	Organ				
	Root Water Content (% FW)	Stem Water Content (% FW)	Leaf Water Content (% FW)	Bract Water Content (% FW)	Whole Plant Water Content (% FW)
CK	87.17 ± 0.44 Aa	90.58 ± 2.44 Aa	84.77 ± 4.18 Aa	83.14 ± 1.90 Aa	86.42 ± 3.83 Aa
T1	84.05 ± 2.54 Ab	87.19 ± 4.42 Aa	81.47 ± 3.36 Aa	82.79 ± 0.81 Aa	83.88 ± 3.66 Ab
T2	81.26 ± 2.67 Bc	85.17 ± 3.58 Ab	78.28 ± 4.64 Bb	80.05 ± 3.45 Ab	81.19 ± 4.43 Bc

Note: The data values are means ± standard deviation (*n* = 3). Different capital letters in the same column denote extremely significant differences (*p* < 0.01), and different small letters in the same column denote significant differences (*p* < 0.05). This applies to the rest of the text hereafter.

**Table 2:** Changes in photosynthetic pigment content in different organs of *B. glabra* ‘Elizabeth Angus’ under different times treatments.

Organ	Treatment	Chl-a (μg·g^−1^ FW)	Chl-b (μg·g^−1^ FW)	Chl (μg·g^−1^ FW)	Car (μg·g^−1^ FW)
Stem	CK	28.46 ± 3.26 Cc	25.18 ± 2.64 Cc	53.64 ± 9.20 Bb	8.49 ± 0.18 Cc
	T1	86.98 ± 1.00 Bb	47.21 ± 0.16 Aa	134.19 ± 0.21 Aa	18.34 ± 0.09 Bb
	T2	93.44 ± 0.72 Aa	34.62 ± 0.37 Bb	128.07 ± 0.52 Aa	29.07 ± 0.17 Aa
Leaf	CK	384.41 ± 5.03 Aa	260.14 ± 6.69 Aa	644.55 ± 6.92 Aa	113.77 ± 0.55 Aa
	T1	269.92 ± 7.45 Bb	119.18 ± 6.86 Bb	389.10 ± 8.09 Bb	58.74 ± 2.05 Bb
	T2	211.56 ± 4.18 Cc	102.36 ± 7.76 Bb	313.91 ± 12.34 Cc	29.88 ± 2.69 Cc
Bract	CK	7.24 ± 1.66 Bb	4.58 ± 1.34 Cc	11.82 ± 3.08 Cc	5.79 ± 0.11 Bbc
	T1	12.32 ± 1.65 Aa	6.05 ± 1.15 Bb	18.38 ± 2.88 Bb	7.08 ± 0.13 Aab
	T2	13.56 ± 1.68 Aa	7.83 ± 0.61 Aa	21.39 ± 1.66 Aa	8.42 ± 0.62 Aa

Note: The data values are means ± standard deviation (*n* = 3). Different capital letters in the same column of same organ denote extremely significant differences (*p* < 0.01), and different small letters in the same column of same organ denote significant differences (*p* < 0.05). This applies to the rest of the text hereafter.

**Table 3:** Changes in chlorophyll fluorescence parameters of *B. glabra* ‘Elizabeth Angus’ under different treatment times.

Types	Index	Treatment		
		CK (Clean Air, 0 μL·L^−1^)	T1 (4 μL·L^−1^ NO_2_ + 8 h/d)	T2 (4 μL·L^−1^ NO_2_ + 24 h/d)
Relative fluorescence parameters (5 indexes)	*Fo*	677.01 ± 11.88 Cc	884.10 ± 123.96 Bb	1206.33 ± 75.20 Aa
	*Fm*	3731.22 ± 312.88 Aa	2769.22 ± 113.97 Bc	3511.78 ± 323.90 Aab
	*Fv*	3054.22 ± 316.36 Aa	1885.11 ± 112.89 Cc	2329.44 ± 376.80 Bb
	*Vi*	0.52 ± 0.03 Cc	0.75 ± 0.01 Bb	0.83 ± 0.03 Aa
	*Vj*	0.86 ± 0.01 Bb	0.90 ± 0.01 Aa	0.88 ± 0.02 ABab
Optical system parameters (6 indexes)	*Fv*/*Fo*	4.52 ± 0.50 Aa	2.13 ± 0.15 Bb	1.95 ± 0.39 Bb
	*Φ*(*Po*) *= Fv*/*Fm*	0.82 ± 0.02 Aa	0.68 ± 0.01 Bb	0.66 ± 0.05 Bbc
	*Φ*(*Eo*)	0.39 ± 0.03 Aa	0.17 ± 0.01 Bb	0.11 ± 0.03 Cc
	*ψ*(*Eo*)	0.48 ± 0.03 Aa	0.26 ± 0.02 Bb	0.17 ± 0.02 Cc
	*Φ*(*Ro*)	0.14 ± 0.01 Aa	0.06 ± 0.01 Bb	0.08 ± 0.05 Bb
	*Φ*(*Do*) *= Fo*/*Fm*	0.19 ± 0.02 Bb	0.32 ± 0.02 Aab	0.35 ± 0.05 Aa
Unit reaction center parameters (3 indexes)	*ABS*/*RC*	2.33 ± 0.16 Bb	3.34 ± 0.07 Aa	3.41 ± 0.45 Aa
	*ETo*/*RC*	0.81 ± 0.15 Aa	0.51 ± 0.12 Bb	0.47 ± 0.16 Bb
	*REo*/*RC*	0.31 ± 0.02 Aa	0.22 ± 0.01 Bc	0.26 ± 0.05 Bb
Blade performance (1 index)	*PI*_(*ABS*)_	1.97 ± 0.45 Aa	0.21 ± 0.02 Bb	0.13 ± 0.06 Bb

Note: *Fo*. initial fluorescence; *Fm*. maximum fluorescence; *Fv*. variable fluorescence; *Vi.* I point relative variable fluorescence; *Vj*. J point relative variable fluorescence; *Fv*/*Fo.* potential quantum yield; *Φ*(*Po*) *= Fv*/*Fm.* maximum quantum yield of Photosystem II; *Φ*(*Eo*) electron transfer quantum yield; *ψ*(*Eo*). the energy ratio of capturing light energy for electron transfer. *Φ*(*Ro*). PSI receptor side terminal electron reduction efficiency; *Φ*(*Do*) *= Fo*/*Fm.* maximum quantum yield of non-photochemical quenching; *ABS*/*RC.* unit reaction center absorbed energy; *ETo*/*RC.* energy used for electron transfer; *REo*/*RC.* light energy transmitted to PSI; *PI*_(*ABS*)._ performance index based on absorbed light energy. Note: The data values are means ± standard deviation (*n* = 3). Different capital letters in the same row denote extremely significant differences (*p* < 0.01), and different small letters in the same row denote significant differences (*p* < 0.05). This applies to the rest of the text hereafter.

**Table 4:** Changes in photosynthetic parameters of *B. glabra* ‘Elizabeth Angus’ under different concentrations of treatments.

Index	Treatment		
	CK	T1	T2
**Pn** (μmol CO_2_·m^−2^·s^−1^) Net photosynthetic rate	7.84 ± 0.53 Aa	5.39 ± 0.73 Bb	3.46 ± 2.20 Cc
Ci (μmol·mol^−1^) Intercellular CO_2_ concentration	184.33 ± 17.44 Bb	175.22 ± 41.35 Bb	243.44 ± 64.89 Aa
**Gs** (mmol H_2_O·m^−2^·s^−1^) Stomatal conductivity	58.00 ± 3.27 Aa	39.33 ± 8.49 Bb	38.33 ± 16.14 Bb
**Tr** (mmol H_2_O·m^−2^·s^−1^) Transpiration rate	2.14 ± 0.14 Aa	1.52 ± 0.31 Bb	1.60 ± 0.67 Bb
**VPD** (kPa) Vapor pressure deficiency	3.79 ± 0.10 Aa	3.47 ± 0.24 Bb	3.84 ± 0.89 Aa
**WUE** (mmol CO_2_·mol) Photosynthetic water use efficiency	3.68 ± 0.43 Aa	3.69 ± 0.91 Aa	2.62 ± 1.11 Bb

Note: Different capital letters in the same row denote extremely significant differences (*p* < 0.01), and different small letters in the same row denote significant differences (*p* < 0.05).

**Table 5:** Classification index component factor load matrix and principal component coefficient.

Index	Principal Component			Index	Principal Component		
	*w*_i1_	*w*_i2_	*w*_i3_		*w*_i1_	*w*_i2_	*w*_i3_
Root volume	0.961	0.050	0.130	*Fo*	0.955	0.172	0.138
Average root diameter	0.962	0.132	0.111	*Fm*	−0.323	0.762	0.494
Main root width	0.955	0.177	0.170	*Vi*	0.980	−0.120	−0.099
Lateral root width	0.948	0.190	0.162	*Vj*	0.669	−0.332	−0.538
Mature leaf L	0.986	−0.010	0.055	*Φ*(*Do*)	0.922	−0.265	−0.147
Mature leaf a	0.992	0.032	0.044	ABS/RC	0.863	−0.332	−0.233
Mature leaf b	0.988	0.053	0.085	Ci	0.417	0.807	−0.351
Tender leaf L	0.984	0.032	0.063	Tr	−0.404	0.687	−0.512
Tender leaf a	0.977	0.118	0.151	WUE	−0.426	−0.750	0.413
Tender leaf b	0.982	0.041	0.088	Eigenvalue λ	17.543	2.893	1.413
Bract L	0.938	0.177	0.132	Variance contribution rate/%	76.273	12.577	6.145
Bract b	0.985	0.095	0.097	Accumulated contribution rate/%	76.273	88.850	94.995
Stem chlorophyll a	0.947	−0.199	−0.120				
Stem total chlorophyll	0.884	−0.322	−0.220	Factor Weight α_i_	0.8029	0.1323	0.0647

**Table 6:** Principal component comprehensive score of different fumigation times of nitrogen dioxide treatment.

Treatment	Principal Components			Composite Score (F)	Composite Score Ranking
	PC1 (F1)	PC2 (F2)	PC3 (F3)		
CK	−5.339	0.840	0.617	−4.135	3
T1-4.0 μL·L^−1^ (8 h/d)	0.746	−1.703	−0.872	0.317	2
T2-4.0 μL·L^−1^ (24 h/d)	4.592	0.864	0.254	3.818	1

**Table 7:** Comprehensive evaluation and ranking of membership functions of *Bougainvillea* under different times fumigation treatment.

Index	CK	T1	T2	Index	CK	T1	T2
Root volume	0.79	0.41	0.07	Stem Chl-a	0.94	0.10	0.01
Average root diameter	0.92	0.53	0.07	Stem total Chl	0.90	0.00	0.07
Main root width	0.90	0.56	0.09	Fo	0.97	0.65	0.15
Lateral root width	0.87	0.53	0.07	Fm	0.87	0.75	0.58
Mature leaf L	0.96	0.39	0.05	Vi	0.88	0.31	0.11
Mature leaf a	0.91	0.39	0.03	Vj	0.81	0.27	0.43
Mature leaf b	0.97	0.45	0.04	Φ(Do)	0.94	0.43	0.33
Tender leaf L	0.91	0.45	0.13	ABS/RC	0.89	0.41	0.38
Tender leaf a	0.93	0.55	0.10	Ci	0.60	0.63	0.41
Tender leaf b	0.92	0.50	0.13	Tr	0.51	0.73	0.71
Bract L	0.95	0.67	0.18	WUE	0.56	0.56	0.37
Bract b	0.99	0.54	0.08	Final Score and ranking	0.8657 1	0.4715 2	0.1982 3

## Data Availability

The data presented in this study are available on request from the corresponding author. The data are not publicly available due to a pending individual invention patent.

## Supplementary Materials

The following supporting information can be downloaded at https://www.mdpi.com/article/10.3390/plants12234028/s1. Figure S1: Changes in leaf morphology indicators of *B. glabra* ‘Elizabeth Angus’ under different fumigation times of treatments; Figure S2: Changes in leaf functional trait indicators of *B. glabra* ‘Elizabeth Angus’ under different fumigation times of treatments; Table S1: Mantel test significance *p*-value and correlation R-value between treatments group and index.
