# Supplementary material for: Acute NO2 Stress Shortens the Median Survival Period of Bougainvillea glabra ‘Elizabeth Angus’ by Disrupting Tissue Structure and Photosynthetic Response Centers
Source: Plants (Basel). 2023 Nov 30;12(23):4028. doi: 10.3390/plants12234028 (PMC10708483; doi:10.3390/plants12234028)
Supplement: Supplementary file 1 [file plants-12-04028-s001.zip › plants-2731963-supplementary.pdf]

Acute NO_2 Stress Shortens the Median Survival Period of *Bougainvillea glabra* 'Elizabeth Angus' by Disrupting Tissue Structure and Photosynthetic Response Centers

Supplementary materials

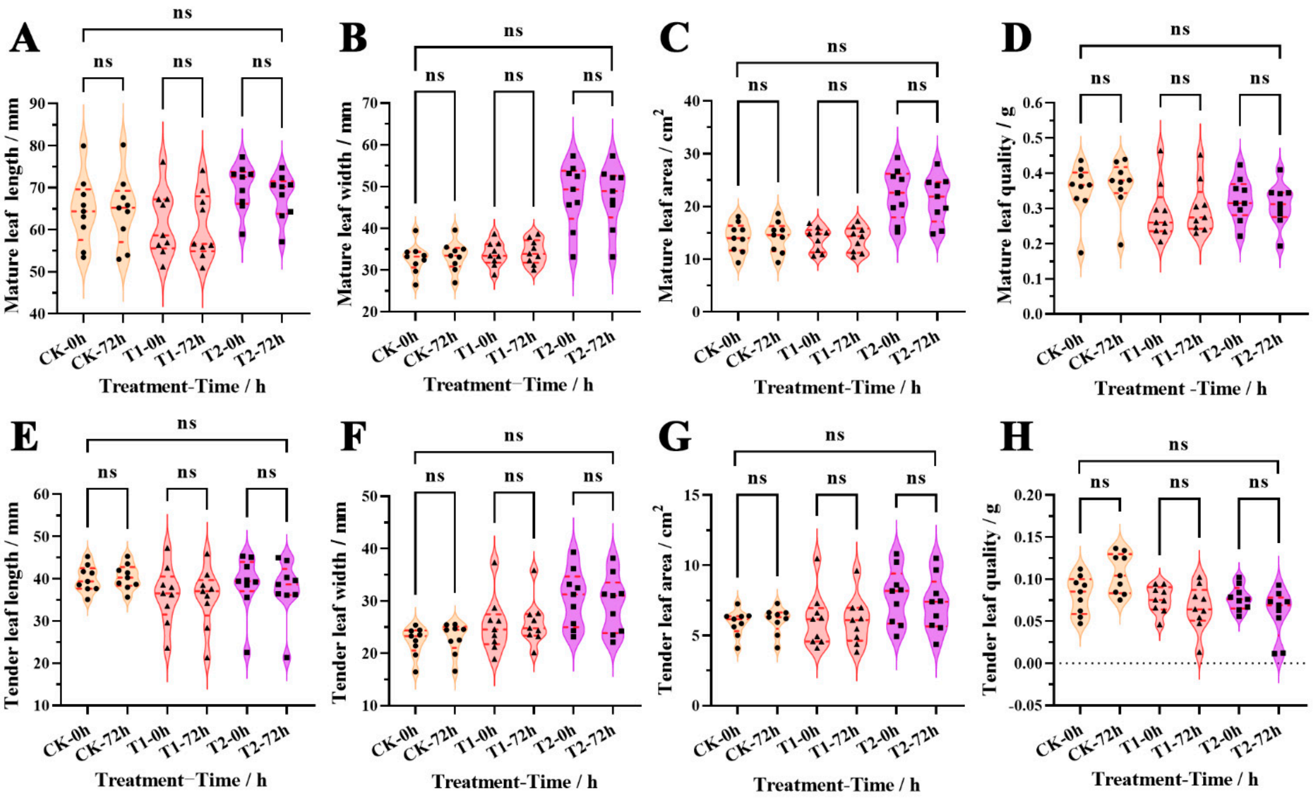

Figure S1. Changes in leaf morphology indicators of *B. glabra* 'Elizabeth Angus' under different fumigation times of treatments. **(A)** Mature leaf length. **(B)** Mature leaf width. **(C)** Mature leaf area. **(D)** Mature leaf quality. **(E)** Tender leaf length. **(F)** Tender leaf width. **(G)** Tender leaf area. **(H)** Tender leaf quality.

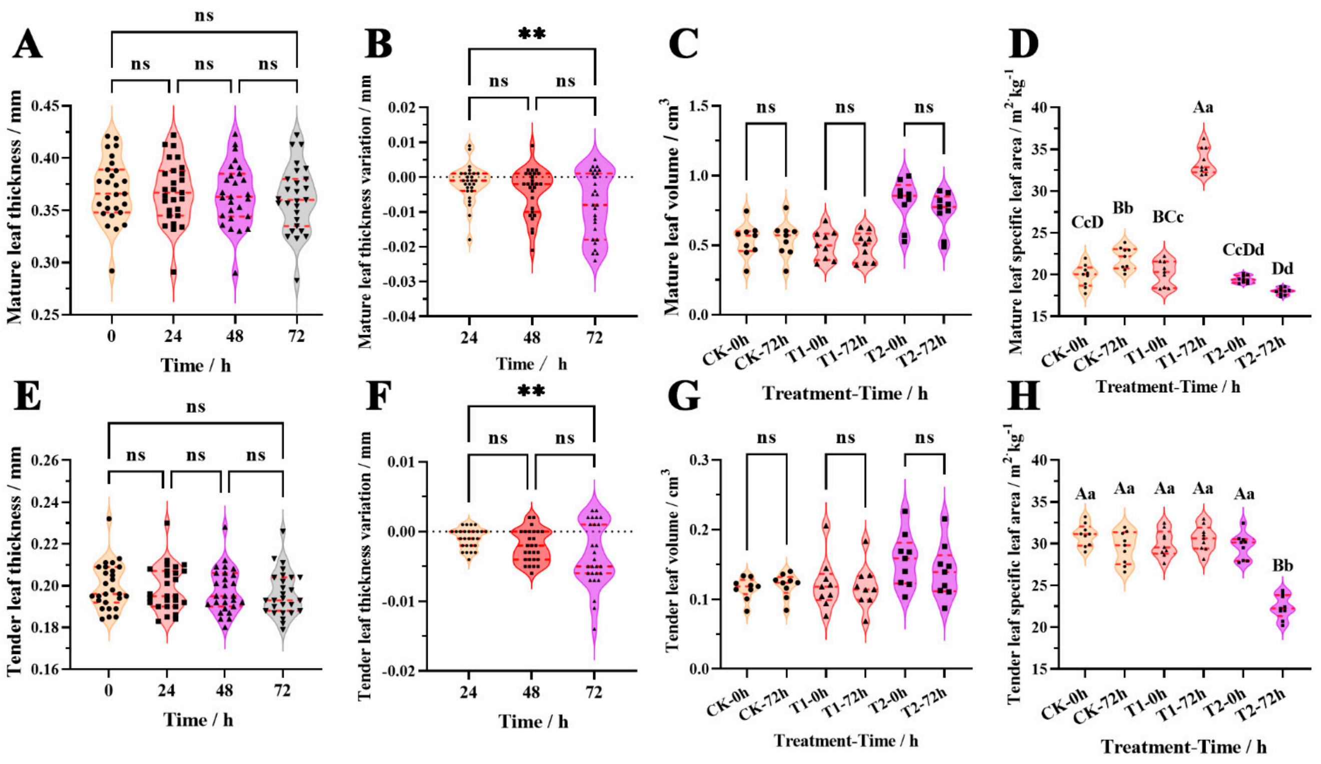

Figure S2. Changes in leaf functional trait indicators of *B. glabra* 'Elizabeth Angus' under different fumigation times of treatments. **(A)** Mature leaf thickness. **(B)** Mature leaf thickness variation. **(C)** Mature leaf volume **(D)** Mature leaf Specific leaf area. **(E)** Tender leaf thickness. **(F)** Tender leaf thickness variation. **(G)** Tender leaf volume **(H)** Tender leaf specific leaf area.

Table S1. Mantel test significance *p*-value and correlation R-value between treatments group and index.

Factor	*p*-value between treatments			Correlation R-value between treatments		
	CK	T1	T2	CK	T1	T2
Total root length	0.405	0	0	-0.318	0.949	0.949
Root surface area	0.197	0	0.001	-0.474	0.949	0.896
Root volume	0.197	1	1	0.474	0	0
Average root	0.145	0.329	0.447	-0.527	-0.369	-0.291
Main root length	0.258	0.893	0.685	0.422	-0.053	0.158
Laternal root length	0.329	0.685	0.329	-0.369	-0.158	-0.369
Main root width	0.787	0.011	0.042	0.105	-0.791	-0.685
Lateral root width	0.145	0.011	0.329	0.527	0.794	-0.369
Mature leaf L chromatism	0.893	0.258	0.197	-0.053	-0.422	-0.474
Mature leaf a chromatism	0.407	0.787	0.946	0.316	-0.105	-0.026
Mature leaf b chromatism	1	0.893	0.787	0	0.053	-0.105
Tender leaf L Chromatism	0.493	0.787	0.143	0.264	0.105	0.529
Tender leaf a Chromatism	0.586	0.168	0.787	0.211	-0.503	-0.105
Tender leaf b Chromatism	0.102	0.786	0.329	0.58	-0.106	0.369
Matuure leaf thickness variation	0.354	0.892	0.405	-0.351	-0.053	-0.318
Mature leaf specific area	0.102	0.068	0.042	0.58	0.632	-0.685
Tender leaf thickness variation	0.374	0.08	0.484	-0.338	-0.611	0.269
Bract L chromatism	0.258	0.258	0.004	0.422	-0.422	0.843
Bract a chromatism	0.493	0.197	0.102	0.264	-0.474	-0.58
Bract b chromatism	0.493	0.493	0.168	0.264	-0.264	0.503
Root water content	0.281	0.29	0.491	-0.404	-0.397	-0.265

Whole plant water	0.893	1	0.786	0.053	0	-0.106
Stem chlorophyll a	0.683	.	0.17	0.159	1	0.5
Leaf chlorophyll a	0.407	0.683	0.254	0.316	0.159	-0.425
Leaf chlorophyll b	0.685	0.893	0.493	-0.158	-0.053	0.264
Bract chlorophyll b	0.283	0.489	0.633	-0.402	-0.266	-0.185
Stem total chlorophyll	0.195	.	.	0.476	1	1
Leaf total chlorophyll	0.407	0.586	0.258	0.316	0.211	-0.422
Leaf carotenoids	0.17	1	0.586	0.5	0	0.211
Fo	0.491	0.787	0.787	-0.265	-0.105	0.105
Fm	0.197	0.893	0.893	-0.474	0.053	0.053
Fv	0.197	0.893	1	-0.474	0.053	0
Vi	0.329	0.023	0.329	0.369	-0.738	0.369
Vj	0.893	0.407	0.329	0.053	-0.316	0.369
Fv/Fo	0.329	0.586	0.586	-0.369	0.211	-0.211
Φ(Po)=Fv/Fm	0.329	0.586	0.786	-0.369	0.211	-0.106
Φ(Eo)	0.893	0.329	0.329	-0.053	0.369	-0.369
ψ(Eo)	0.893	0.023	0.145	-0.053	0.738	-0.527
Φ(Ro)	0.685	0.407	0.145	-0.158	0.316	-0.527
Φ(Do)=Fo/Fm	0.329	0.586	0.586	0.369	-0.211	0.211
ABS/RC	0.685	0.734	0.329	0.158	0.132	0.369
ETo/RC	0.023	0.685	0.493	-0.738	-0.158	-0.264
REo/RC	0.288	0.893	0.493	-0.399	0.053	-0.264
PI（ABS)	0.493	0.685	0.258	-0.264	0.158	-0.422
Pn	0.491	0.581	0.038	0.265	-0.214	-0.694
Ci	1	0.535	0.682	0	0.239	0.159
Gs	0.683	0.681	0.633	-0.159	0.16	-0.185
Tr	0.839	0.583	1	-0.079	0.213	0
VPD	0.258	0.102	0.946	-0.422	-0.58	0.026
WUE	0.893	0.1	0.633	0.053	-0.582	-0.185
